# Growth-stage dependent changes of leaf chlorophyll content as a proxy for photosynthetic capacity in maize

**DOI:** 10.3389/fpls.2026.1758994

**Published:** 2026-03-19

**Authors:** Wenjing Wang, Bin Chen, Tao Qi, Lihua Hao, Taimiao Fu, Jiarui Zhang, Yue Li

**Affiliations:** 1 School of Earth Science and Engineering, Hebei University of Engineering, Handan, China; 2 Key Laboratory of Ecosystem Network Observation and Modeling, Institute of Geographic Sciences and Natural Resources Research, Chinese Academy of Sciences, Beijing, China; 3 National Ecosystem Science Data Center, Beijing, China; 4 School of Landscape and Ecological Engineering, Hebei University of Engineering, Handan, China; 5 School of Water Conservancy and Hydropower, Hebei University of Engineering, Handan, China

**Keywords:** flowering stage, leaf nitrogen content, leaf photosynthetic capacity, leaf photosynthetic pigments, maize

## Abstract

Accurately quantifying leaf photosynthetic capacity and its seasonal dynamics is critical for simulating gross primary productivity of farmland ecosystems. Previous studies found that leaf chlorophyll (Chl) content was a better proxy for leaf photosynthetic capacity than leaf nitrogen (N) content for C3 crops. However, the relationships of leaf photosynthetic capacity with leaf Chl and N contents for C4 crops (i.e. maize) remain unclear. To address this question, we measured leaf N, Chl, carotenoid (Car) contents, and leaf photosynthetic capacity quantified by the maximum PEP carboxylation rate (V_pmax_), maximum carboxylation rate (V_cmax_) and maximum electron transport rate (J_max_) weekly for a summer maize cropland in North China from the tillering stage to maturity. The results revealed that leaf photosynthetic capacity and leaf biochemistry parameters exhibited distinct dynamic patterns, increasing during vegetative stages and declining during reproductive growth. The relationships between photosynthetic capacity and biochemical variables (i.e., leaf N, Chl, Car contents) differed pre- and post-flowering. The strongest correlation was observed between the maximum carboxylation rate at 25 °C (V_cmax25_) and leaf Chl content by area (Chl_area_) (R^2^ = 0.81) before flowering, followed by N content by area (N_area_) and Car content by area (Car_area_) (R^2^ = 0.78 and 0.62, respectively). The explanatory power of Chl_area_, N_area_, and Car_area_ for V_cmax25_ declined to varying degrees after flowering (R^2^ = 0.74, 0.50, and 0.52, respectively). The Variable Influence on the Projection (VIP) scores and loading analyses also showed that the relationship between V_cmax25_ and biochemical variables exhibited growth-stage dependency. Multiple linear regression analysis revealed that the combination of leaf N_area_, Chl_area_, and Car_area_ had higher accuracy in estimating V_cmax25_ (pre-flowering: R^2^ = 0.94, RMSE = 1.50 μmol·m^−2^·s^−1^; post-flowering: R^2^ = 0.78, RMSE = 4.44 μmol·m^−2^·s^−1^) than individual leaf traits. However, the estimation accuracy of V_cmax25_ based on leaf N_area_, Chl_area_, and Car_area_ showed R^2^ value only 0.13 (pre-flowering) and 0.04 (post-flowering) higher than that of leaf Chl_area_ alone. These findings imply that integrating leaf Chl with growth stage information could enhance the estimation of leaf photosynthetic capacity in C4 crops.

## 1 Introduction

Farmland ecosystems comprise 40% of global terrestrial ecosystems (Hu et al., 2020; Liu X. et al., 2024; Zabel et al., 2019). They play a critical role in global climate change and carbon cycling (Liu et al., 2022; Pang et al., 2025; Robertson et al., 2000). Accurately quantifying the carbon cycle of farmland ecosystems is essential for relieving climate change and ensuring sustainable crop yields (Liu L. et al., 2024). Terrestrial Biosphere Models (TBMs) are critical tools for estimating current and projecting future regional and global terrestrial carbon budgets (Huntzinger et al., 2013), yet their predictions remain highly uncertain (Bonan et al., 2019; Hardouin et al., 2022; MacBean et al., 2022). This uncertainty is resulting from the poor parameterizations of leaf photosynthetic capacity that regulate photosynthesis rate simulations in these models (Lombardozzi et al., 2015; Rogers et al., 2017a, 2017b).

Maize (*Zea mays L.*) is a core component of farmland ecosystems, accounting for 25% of the global cropland and contributing to over 30% of the total annual global crop yield (Kaushal et al., 2022; Liu et al., 2021). Maize is a C4 plant, of which the photosynthesis is achieved through the collaboration of mesophyll and bundle sheath cells and operates an additional CO_2_ concentrating mechanism (CCM), known as the C4 cycle (Von Caemmerer, 2000; Raghavendra and Sage, 2010). CO_2_ is initially fixed in mesophyll cells by phosphoenolpyruvate carboxylase (PEPc) into C4 acids, which are then decarboxylated in bundle sheath cells to supply CO_2_ for Rubisco (Raghavendra and Sage, 2010). The three-carbon compound (pyruvate) formed after decarboxylation returns to the mesophyll cells, where it is re-phosphorylated into phosphoenolpyruvate, thereby completing the cycle (Hatch, 1987; Von Caemmerer, 2000).

Although the distinct intercellular collaboration of C4 crops makes their photosynthesis more intricate, the C4 photosynthesis model has a similar structure to the widely used model of C3 photosynthesis by Farquhar et al. (1980) (Von Caemmerer, 2021). C4 photosynthesis can be limited either by the enzymatic rates of PEPc and Rubisco or by the capacity of chloroplast electron transport, which supports the regeneration of PEP and ribulose bisphosphate (RuBP). The actual CO_2_ assimilation rate is the minimum of the enzyme limited and electron transport limited rate. Thus, the leaf photosynthesis rate (A) of C4 species is described as the minimum of two potential rates: the enzyme-limited rate (Ac) and the electron transport-limited rate (Aj) (Von Caemmerer, 2021). Ac depends on the maximum carboxylation rate (V_cmax_) and maximum PEP carboxylation rate (V_pmax_), adjusted for mitochondrial respiration and CO_2_ leakage. Aj is influenced by the maximum electron transport rate (J_max_). Therefore, V_cmax_, V_pmax_, and J_max_ are critical parameters governing the leaf photosynthetic capacity of C4 plants.

In the A-C_i_ response curve of C4 plants, the light-saturated leaf CO_2_ assimilation rate increases with increasing intercellular CO_2_ concentration (C_i_) until it reaches a plateau. This plateau marks a shift in photosynthesis from an initial phase limited by PEPc activity to a saturated phase dominated by Rubisco activity or electron transport limited rates (Von Caemmerer, 2021). The PEPc activity often limits photosynthesis under low light or low CO_2_ conditions due to insufficient C4 acid production, which restricts CO_2_ delivery to Rubisco. Rubisco activity or electron transport rates limited photosynthesis when the light and CO_2_ supply is sufficient (Furbank et al., 2000). Under field conditions, this transition for C4 crops occurs at a C_i_ of approximately 120 μmol·mol^−1^, beyond which photosynthesis achieves CO_2_ saturation (Leakey et al., 2006). Pignon and Long (2020) analyzed 842 sets of A-C_i_ response data from 49 C4 species and found that the CO_2_ saturation point was highly consistent across various C4 species and remained unaffected by growth conditions under sub-ambient or elevated atmospheric CO_2_ concentrations. Therefore, in both current and future high CO_2_ atmospheric environments, C4 photosynthesis generally operates within the CO_2_-saturated region and is primarily determined by Rubisco activity or electron transport-limited rates.

Typically, a strong correlation has been found between Rubisco activity and the rate of photosynthesis at high CO_2_ concentrations in maize (Usuda, 1984; Sonawane et al., 2017). Recent studies have consistently suggested that with rising atmospheric CO_2_, Rubisco has become the greatest limitation on light-saturated leaf CO_2_ assimilation rates (A_sat_) in C4 crops (Pignon and Long, 2020; Salesse-Smith et al., 2025). Additionally, the non-steady-state metabolic modeling and measurements show Rubisco activity having significant control over the efficiency of photosynthesis in fluctuating light (Wang et al., 2021). Meanwhile, Von Caemmerer (2021) revised the parameters of the steady-state model of C4 photosynthesis, showing that the electron transport rate becomes the dominant limiting factor for CO_2_ assimilation at 25 °C, high CO_2_ concentrations, and high irradiance. This balance can vary with growth conditions or species. Therefore, the maximum carboxylation rate at 25^°^C (V_cmax25_), a key indicator of the inherent catalytic potential of the Rubisco enzyme, not only reflects leaf photosynthetic capacity but also serves as a central variable linking leaf biochemical mechanisms with model simulations.

The representation of V_cmax25_ in TBMs still has considerable uncertainty due to its pronounced spatial and temporal variations (Wang et al., 2022; Zhang et al., 2024). Large seasonal and spatial variations in V_cmax25_ have also been observed within each PFT, especially for diverse crop rotation systems (He et al., 2019). To investigate the temporal and spatial variations of V_cmax25_, a relatively simple and widely adopted method is to utilize its correlation with leaf nitrogen (N) content, as leaf N is an integral component of the Rubisco enzyme in C3 and C4 species (Schmitt and Edwards, 1981; Sage et al., 1987; Makino et al., 2003; Tazoe et al., 2006). Therefore, most studies have focused on the correlation between leaf N and V_cmax25_ (Evans, 1989; Kattge et al., 2009; Walker et al., 2014; Crous et al., 2018; Miner and Bauerle, 2019; Wang et al., 2022). However, some studies have demonstrated that the use of leaf N as a proxy for V_cmax25_ has various limitations. First, the leaf N is partitioned into photosynthetic and non-photosynthetic nitrogen components (Takashima et al., 2004; Onoda et al., 2004). The presence of non-photosynthetic nitrogen complicates the accurate assessment of the relationship between photosynthetic nitrogen and V_cmax25_. Second, the accurate retrieval of leaf N from remote sensing data remains challenging (Knyazikhin et al., 2013), hampering the mapping of V_cmax25_ at regional or global scale.

Recently, the relationship between V_cmax25_ and leaf chlorophyll (Chl) content has received increasing attention. Emerging evidence has demonstrated that leaf Chl can serve as a more reliable proxy for V_cmax25_ than leaf N (Croft et al., 2017; Houborg et al., 2013, 2015; Li et al., 2023; Luo et al., 2018, 2019; Qian et al., 2021). These findings supported the hypothesis of coordinate regulation of photosynthetic components including light harvesting, photochemical, and biochemical components. In addition, it is a significant advantage to take Chl instead of leaf N as a proxy for leaf photosynthetic capacity, due to the distinct optical absorption characteristics of Chl, enabling rapid and accurate retrieval from remote sensing data (Croft et al., 2013; Houborg et al., 2013; Xu et al., 2019). Besides leaf Chl, leaf carotenoids (Car) content plays a crucial role in photosynthesis, especially in its complementary light collection ability at wavelengths where the absorption efficiency of chlorophyll is limited (Chen, 2014; Gitelson et al., 2006; Li et al., 2025). Leaf Car extend the wavelength range of light capture, which can promote photosynthesis (Frank and Cogdell, 1993).

Although the positive relationship between V_cmax25_ and leaf Chl was observed in some studies, the strength and slope of this relationship can vary depending on species, leaf age, and crop growth stage (Li et al., 2022; Qian et al., 2025; Wang et al., 2020). The growth stage of crops is a crucial factor in the relationship between leaf Chl and V_cmax25_ (Li et al., 2022; Qian et al., 2025). Compared to the relatively smooth phenological processes of natural ecosystems, crops exhibit distinct growth stages accompanied by rapid physiological transitions. Lu et al. (2020) pointed out that the current Rubisco-based semi-mechanistic model still cannot simulate V_cmax25_ well in some cases due to without consideration of seasonal change in Rubisco. Li et al. (2022) indicated that leaf Chl_area_ showed significantly different linear relationships with V_cmax25_ pre- and post-flowering while N_area_ did not for both winter wheat and paddy rice. Therefore, it is necessary to investigate the growth-stage dependent changes of relationships between Chl and leaf photosynthetic capacity.

Currently, the understanding of the relationship between leaf photosynthetic pigments and photosynthetic capacity is still mainly derived from C3 plants, whereas the relationship has not been fully investigated in C4 plants. Thus, this study tried to explore the relationships of leaf photosynthetic capacity with leaf N and photosynthetic pigments across different growth stages based on synchronous field measurements of leaf biochemical and photosynthetic parameters for maize. The specific objectives of this study were (1) to characterize the seasonal dynamics of leaf N content, photosynthetic pigments and photosynthetic capacity for maize, (2) to investigate how the relationships of photosynthetic capacity with leaf N content or photosynthetic pigments changed with growing stages of maize, (3) to identify the best proxy for leaf photosynthetic capacity in maize.

## 2 Materials and methods

### 2.1 Field site description

**Figure 1:**
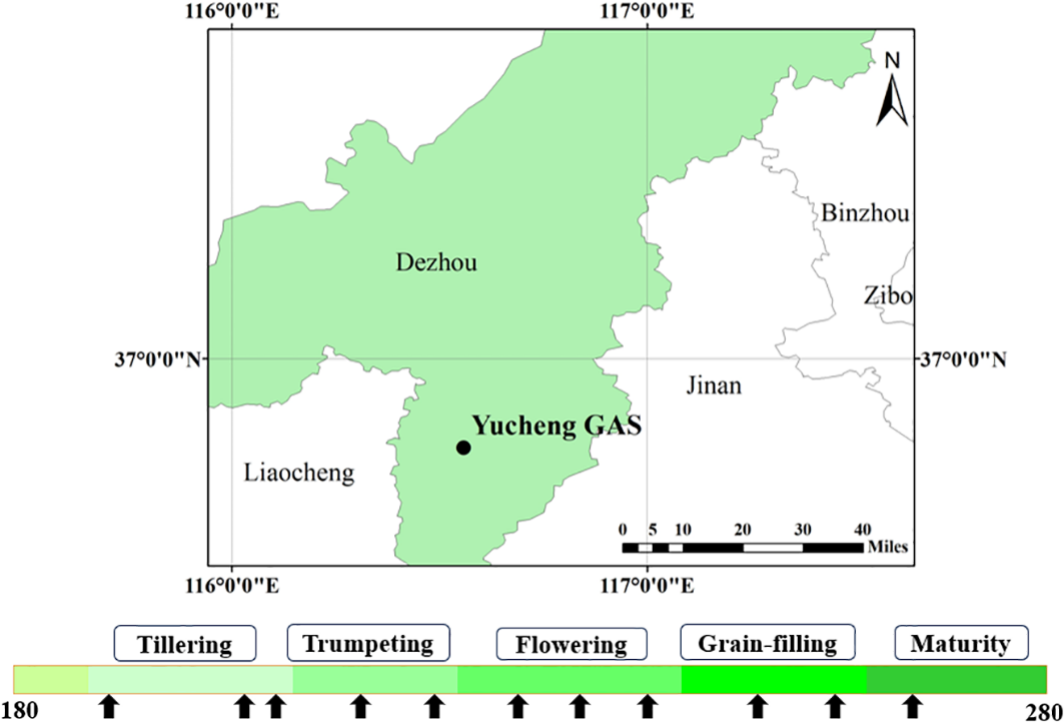
The location of the study site (the upper figure) and the day of year (DOY) for different growth stages and the measurements at the site (the lower figure). On the lower figure, colors represent durations of different growth stages and black arrows indicate each observation date.

The experiments were conducted in Yucheng Agro-ecosystem National Observation and Research Station, Ministry of Science and Technology, located in Dezhou city, Shandong province (36^°^50′ N, 116^°^34′ E, 23 m a.s.l.) within the North China Plain (Zhao et al., 2018) (**Figure 1**). This site is situated in warm temperate, semi-humid monsoon climate zone and represents a typical maize-growing region in China. The average air temperature was 14.5 °C in 2021. The maize growing season spanned from June to October in 2021. Most of the rainfall occurred from June to August, and the average daily precipitation was 70–80 mm. The soil is mainly composed of fluvo-aquic and saline fluvo-aquic soil in this area. The field capacity is 0.27 m^3^ m^-3^, and the soil total nitrogen mass fraction is 0.64 g·kg^-1^ (Gong et al., 2020).

Our measurements were conducted in a 5 m × 5 m plot near the flux tower during the growing season of the summer maize from the day of the year (DOY) 190 (July 7) to 267 (September 23) in 2021 (**Figure 1**). The measurement period encompassed five critical growth stages: tillering stage (DOY = 190, 203, 206), trumpeting stage (DOY = 214, 221), flowering stage (DOY = 228, 235, 242), grain-filling stage (DOY = 252, 260), and maturity stage (DOY = 267). Measurements were conducted around every 7 to 10 days.

### 2.2 Measurements of leaf-level gas exchanges

Leaf-level gas exchanges were measured on the fully expanded top leaves of 2–3 uniformly grown plants of summer maize each time during DOY 190 to 267 in 2021, using a portable photosynthesis system (LI-6400; Li-COR, Inc., Lincoln, NE, USA). The carbon dioxide (CO_2_) response curves (A-C_i_ curves) were produced under conditions of saturated photosynthetic photon flux density (PPFD) (1500 µmol·m^-2^·s^-1^) and stepwise CO_2_ concentration gradient of 380, 300, 200, 100, 50, 380, 600, 800, 1000, and 1200 µmol CO_2_ mol^-1^ air. Prior to the logging measurements, the leaves were acclimated for 20 minutes under the condition of PPFD of 1500 µmol·m^-2^·s^-1^, relative humidity of 60%-80%, ambient temperature, and CO_2_ concentration of 380 µmol·mol^-1^ in a 2×3 cm^2^ leaf chamber. During the measurement, the leaf chamber was kept as close to 25 °C as possible and relative humidity kept between 60% and 80%.

The photosynthetic parameters (V_pmax_, V_cmax_, and J_max_) were derived from the A-C_i_ curves fitted using the “minpack.lm” and “Metrics” R Packages (https://zhujiedong.quarto.pub/photosynthesis-school-2023/#/c4-%E6%A4%8D%E7%89%A9%E6%89%8B%E5%86%99%E4%BB%A3%E7%A0%81%E5%8F%8A%E4%BD%9C%E5%9B%BE). The estimation method is based on the C4 photosynthesis model, which was described in detail in the supplementary material. The fitted V_pmax_, V_cmax_, and J_max_ parameters were scaled to a reference temperature of 25 °C using modified Arrhenius equation (Equation 1; **Table 1**) to facilitate comparability with existing datasets (Massad et al., 2007).

**Table 1:** Parameters values referring to the temperature responses of leaf photosynthetic capacity.

Parameter	E_a_	H_d_	ΔS
V_cmax_	67294	144568	472
J_max_	77900	191929	627
V_pmax_	70373	117910	376

(1) $$f\left(T_{k}\right)\;=\;k_{25}\exp[\frac{E_{a}(T_{k}-298)}{\left(298RT_{k}\right)}]\frac{1+\exp(\frac{298\Delta S-H_{d}}{298R})}{1+\exp(\frac{T_{k}\Delta S-H_{d}}{T_{k}R})}$$

where f(T_k_) and k_25_ represent temperature-dependent values at leaf temperature (T_k_) and 25 °C, respectively. E_a_ refers the activation energy (and is analogous to parameter E_a_ in the Arrhenius function), H_d_ indicates the deactivation energy and ΔS is known as an entropy factor. R is the molar gas constant (8.314 J·mol^-1^·K^-1^).

Additionally, to illustrate how the photosynthetic rate of maize leaves is limited by different parameters based on the fitted values, photosynthetic rates limited by PEPc (Acp), Rubisco (Acr), and electron transport (Aj) were simulated using the estimated parameters within a C4 biochemical model framework. The simulation equations were described in detail in the **Supplementary Material**.

### 2.3 Leaf biochemistry measurements

Leaf nitrogen, chlorophyll, carotenoid content, and leaf mass per unit area (LMA) were measured for the same leaves as leaf-level gas exchange measurements. The leaf samples were excised from maize plants, placed in labeled paper bags, and then stored in an ice box. These samples were quickly moved to the laboratory.

For the measurement of leaf chlorophyll and carotenoid content, two leaf disks were punched from a sampled leaf by a cork borer with known area (6 square centimeters). After punching, 12 cm^2^ sub-sample was taken from the remaining parts of the leaves and used for measuring leaf nitrogen content. The leaf chlorophyll and carotenoid were extracted by homogenate in a pre-chilled mortar with quartz sand, calcium carbonate, and 2–3 ml of 95% ethanol. The homogenate was filtered through filter paper, and the filtrate was diluted to a final volume of 25 ml using 95% ethanol. The absorbance values of the dissolving solution were measured at 665, 649, and 470 nm using a spectrophotometer (UV-2550, Shimadzu Inc., Japan). Leaf chlorophyll a (Chl_a_), chlorophyll b (Chl_b_), and carotenoid (Car) concentrations were calculated according to Fargasová and Molnárová (2010). The dissolving solution was divided into three portions for measurement, with the average of three replicates representing the pigment concentrations.

For the measurement of leaf N content, leaf samples were dried in an oven at 65 °C for 48 hours until a constant weight. After weighting the dry weight, the samples were ground to powder using a high-speed oscillation ball mill (MM400, RETSCH, Germany) to ensure uniform particle size for subsequent elemental analysis. The resulting powder was sealed in labeled plastic bags for nitrogen content per unit mass (N_mass_) measurement with a CN elemental analyzer (Elementar Analyzer system, Hanau, Germany) (Wang et al., 2016). Leaf nitrogen content by area (N_area_) was calculated based on leaf nitrogen content per unit mass, dry weight, and leaf area (Equation 2). It can be expressed as:

(2) $$N_{area}\;=\;\frac{N_{mass}W_{d}}{A}10$$

where N_mass_ is the leaf nitrogen content per unit mass (mg·g^-1^), W_d_ is the leaf dry weight (g), and A is the leaf area (cm^2^).

LMA was calculated using leaf dry weight and leaf area (Equation 3), it can be expressed as:

(3) $$LMA\;=\;\frac{W_{d}}{A}$$

### 2.4 Statistical analyses

For correlation analysis, we used Pearson’s correlation coefficient (R) through R software (version 4.4.1) to quantify the strength of relationships among these variables. The value of R is between -1 and 1, with larger absolute values indicating stronger positive (R > 0) or negative (R< 0) correlations. To indicate the seasonal variations in the regulation of leaf photosynthetic capacity by biochemical parameters, the relationships between leaf photosynthetic capacity (i.e., V_pmax25_, V_cmax25_, and J_max25_) and leaf biochemical parameters (i.e., leaf N, Chl, and Car content) were evaluated by simple linear regressions at the pre- and post-flowering stages, respectively. The relationships were evaluated using the coefficient of determination (R^2^) and statistical significance of the regression slopes (α = 0.01). We used the statistical package Origin 2024 to conduct simple linear regressions. To clearly quantify the relative contributions of leaf biochemical variables to V_cmax25_, we adopted variable importance in projection scores (VIP scores) and loading analysis of Partial Least Squares Regression (PLSR) (Wang et al., 2021; Wold et al., 2001). The VIP is a widely used multivariate statistical method based on PLSR, which quantifies the relative contributions of different independent variables to dependent variables. The high absolute values of loading and VIP scores indicate a high contribution to V_cmax25_. The VIP scores and loading analyses were computed using Origin 2024. Multiple linear regression models were constructed to explore the effects of leaf N, Chl, and Car contents on variations in leaf photosynthetic capacity during the pre- and post-flowering stages (Li et al., 2023). The performances of different leaf trait combinations were estimated using coefficient of determination (R^2^) and Root Mean Square Error (RMSE) between different leaf trait combinations and leaf photosynthetic parameters. We used SPSS version 24.0 (SPSS Inc. Chicago, IL, USA) to perform multiple linear regression analyses in our study.

## 3 Results

### 3.1 Seasonal changes in leaf biochemistry and photosynthetic parameters

**Figure 2:**
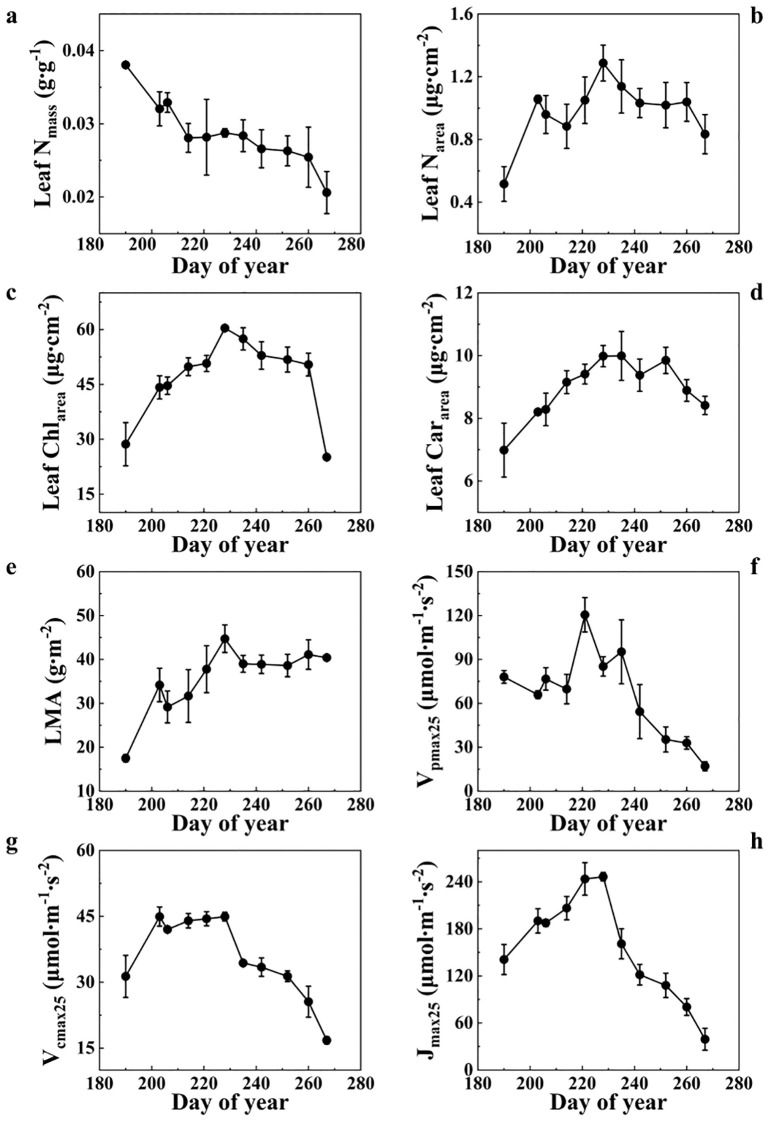
Seasonal changes in 2021 for **(a)** leaf nitrogen content by mass, **(b)** leaf nitrogen content by area, **(c)** leaf chlorophyll content (Chl_area_), **(d)** leaf carotenoid content (Car_area_), **(e)** leaf mass per unit area (LMA), **(f)** the maximum rate of PEP carboxylation rate of at 25 °C (V_pmax25_), **(g)** leaf maximum carboxylation rate at 25 °C (V_cmax25_) and **(h)** leaf maximum electron transport rate at 25 °C (J_max25_), for the measured maize.

The photosynthetic parameters and leaf biochemistry parameters of summer maize leaves exhibited distinct dynamic patterns throughout the growing season in 2021 (**Figure 2**). Leaf nitrogen (N) was expressed based on mass (N_mass_; **Figure 2a**) and area (N_area_; **Figure 2b**), respectively. They exhibited different seasonal variations. N_mass_ showed a maximum value of 0.038 g·g^-1^ on DOY 190 (the tillering stage) and then it rapidly decreased to 0.028 g·g^-1^ on DOY 214 (the trumpeting stage), following by stabilization during the flowering and the grain-filling stages and a drop in the maturity stage (**Figure 2a**). LMA increased persistently from 17.5 g·m^−2^ at the tillering stage to 40.42 g·m^−2^ at the flowering stage and remained stable during the rest of the growing season (**Figure 2e**). The N_area_ trend was adjusted by variations in leaf mass per unit area (LMA), with lower LMA values at the start of the growing season offsetting the higher N_mass_ values in early season. N_area_ increased rapidly during the tillering stage, then increased gradually and peaked at the flowering stage (1.29 g·m^-2^). Then, N_area_ declined gradually until the maturity stage (0.73 g·m^-2^) (**Figure 2b**).

Moreover, photosynthetic parameters displayed similar seasonal variations to Chl_area_ and Car_area_ (**Figures 2c, d, f–h**). Leaf Chl_area_ and Car_area_ increased gradually during the start of the season, peaked at the flowering stage, and then declined gradually after the flowering stage from 60.39 (DOY 228) to 50.42 μg·cm^-2^ (DOY 260) and from 9.99 to 8.89 μg·cm^-2^, respectively. After maturity, the values decreased rapidly to 25.12 μg·cm^-2^ and 8.41 μg·cm^-2^, respectively (**Figures 2c, d**). Moreover, smaller seasonal changes were observed in Car_area_ compared to Chl_area_, particularly at the maturity stage. V_cmax25_ and J_max25_ increased rapidly and reached their peak at the flowering stage, and then declined rapidly from 44.92 (DOY 228) to 16.78 μmol·m^−2^·s^−1^ (DOY 267) and from 246.50 to 39.26 μmol·m^−2^·s^−1^, respectively (**Figures 2g, h**). Notably, V_pmax25_ values decreased slightly over the first four measurement periods from 78.05 μmol·m^−2^·s^−1^ to 69.74 μmol·m^−2^·s^−1^, and then increased rapidly and reached its peak on DOY 221. Then, V_pmax25_ declined rapidly to 17.05 μmol·m^−2^·s^−1^ during the rest of the growing season (**Figure 2f**). Furthermore, the seasonal variations in maize photosynthetic rate limited by different parameters are presented in the **Supplementary Materials** (**Supplementary Figure 1**).

### 3.2 Correlations between leaf biochemical parameters and photosynthetic capacity

**Figure 3:**
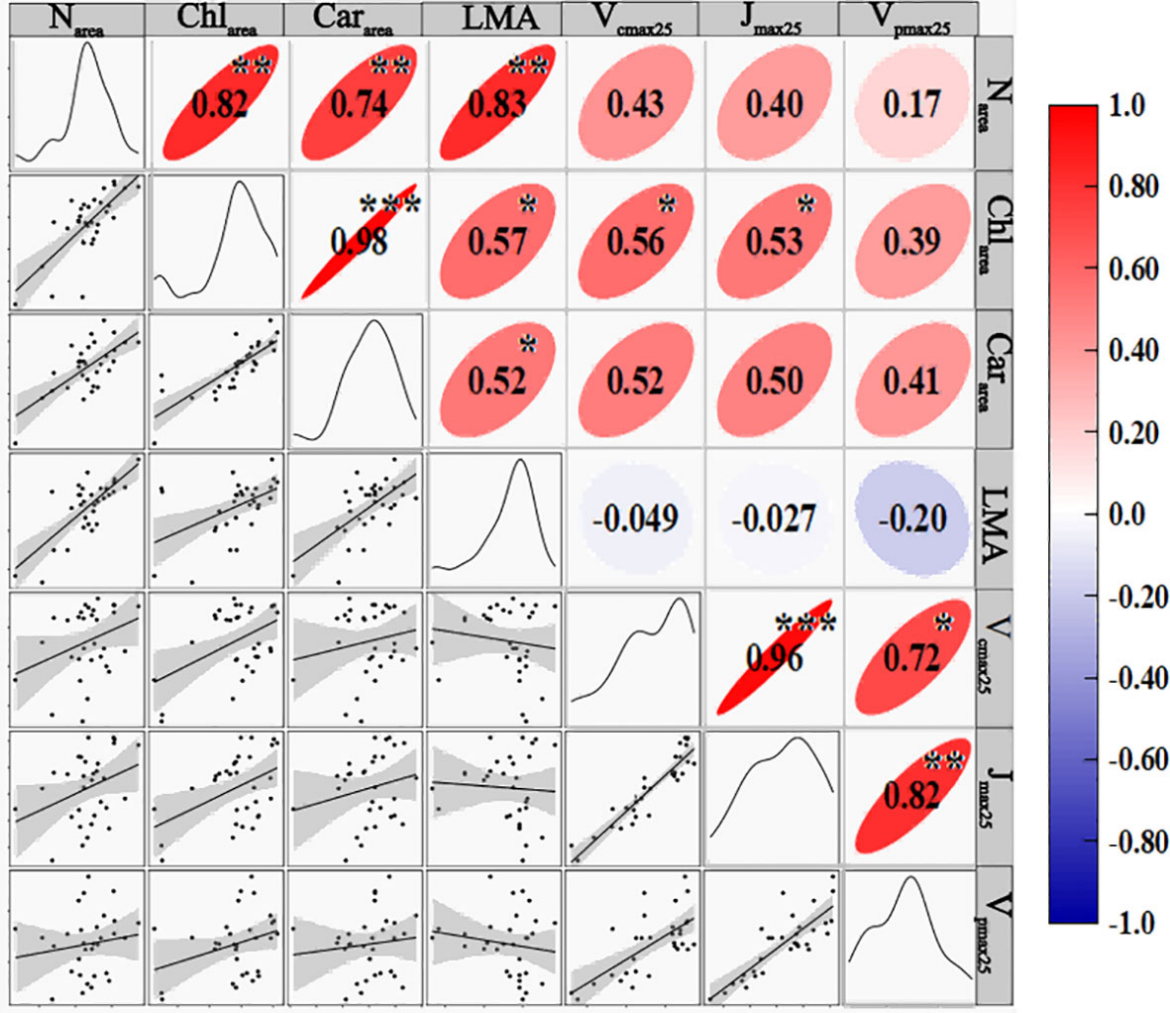
Correlation coefficients of leaf biochemical properties and photosynthetic parameters for the measured maize in 2021. *, **and*** represent p< 0.1, p< 0.01, and p< 0.001, respectively.

**Figure 3** demonstrated the correlation coefficients (R) between leaf photosynthetic capacity (V_cmax25_, J_max25_, and V_pmax25_) and biochemical parameters (N_area_, Chl_area_, Car_area_, and LMA) for the summer maize. N_area_, Chl_area_ and Car_area_ all showed positive correlations with both V_cmax25_ and J_max25_, respectively. The strongest correlation was observed with Chl_area_ (R = 0.56 and 0.53), followed sequentially by Car_area_ (R = 0.52 and 0.50) and N_area_ (R = 0.43 and 0.40) (**Figure 3**). V_pmax25_ was also positively correlated with leaf biochemical parameters (N_area_, Chl_area_, and Car_area_), with correlation coefficients of 0.17, 0.39, and 0.41, respectively. Compared to other parameters, LMA showed almost no significant correlation with leaf photosynthetic capacity. Additionally, significant correlations existed among leaf N_area_, Chl_area_, and Car_area_, and highly significant correlations existed among the photosynthetic parameters (V_cmax25_ Vs. J_max25_, V_cmax25_ Vs. V_pmax25_, and J_max25_ Vs. V_pmax25_) (**Figure 3**).

### 3.3 Growth-stage dependent correlations of leaf photosynthetic capacity with leaf biochemical variables

**Figure 4:**
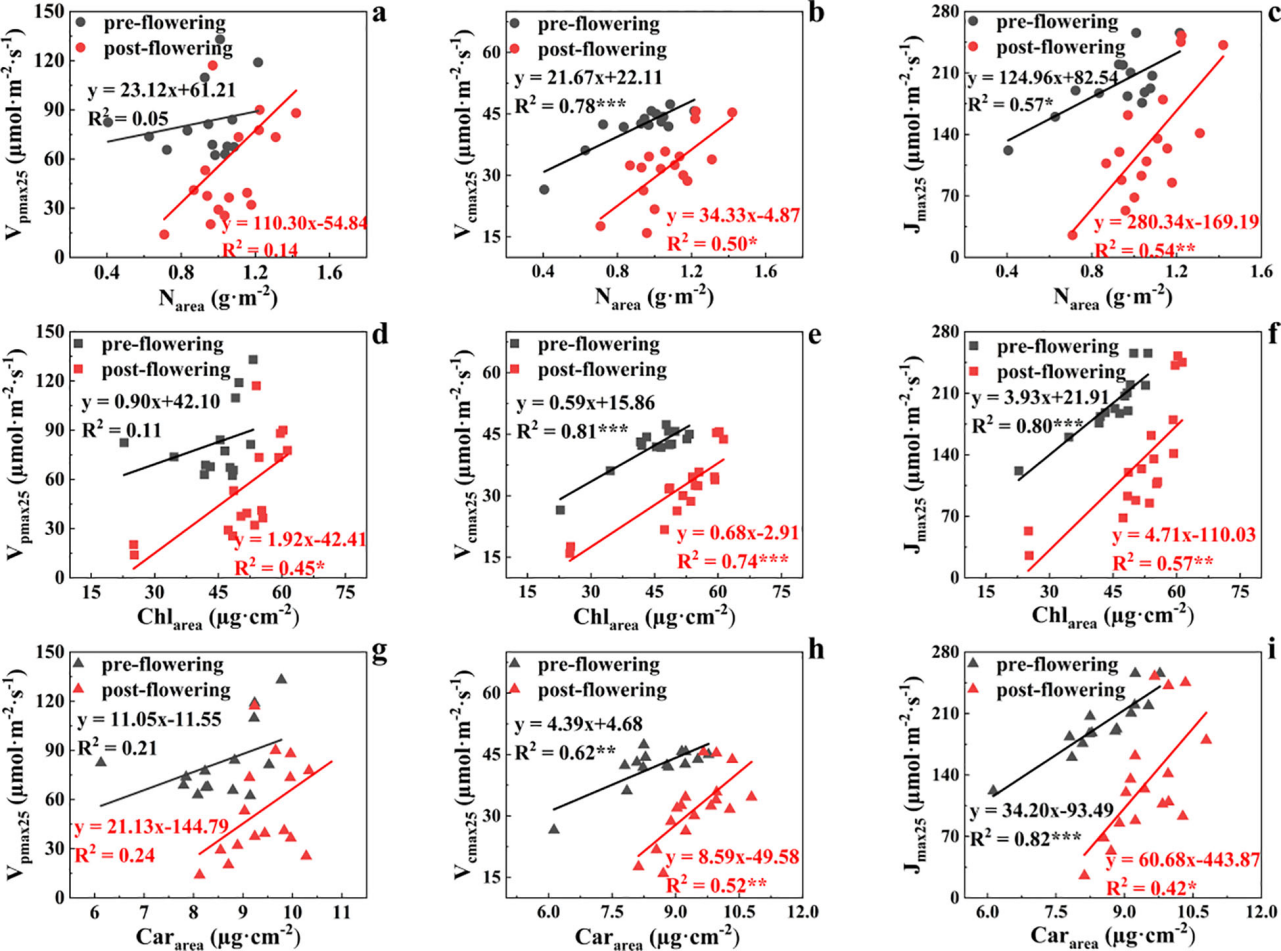
Correlations between N_area_ and V_pmax25_ **(a)**, N_area_ and V_cmax25_ **(b)**, between N_area_ and J_max25_ **(c)**, Chl_area_ and V_pmax25_ **(d)**, between Chl_area_ and V_cmax25_ **(e)**, between Chl_area_ and J_max25_ **(f)**, Car_area_ and V_pmax25_ **(g)**, between Car_area_ and V_cmax25_ **(h)**, between Car_area_ and J_max25_ **(i)**. The grey and red points represent the measurements at pre- and post- flowering stages, respectively. *, ** and *** represent p< 0.01, p< 0.001, and p< 0.0001, respectively.

The results in **Figure 4** showed the relationships between photosynthetic parameters (i.e., V_pmax25_, V_cmax25_, and J_max25_) and leaf biochemical variables (i.e., N_area_, Chl_area_, and Car_area_) and their correlations differed pre- and post-flowering. The correlation of V_pmax25_ with leaf biochemical parameters was weaker than that of V_cmax25_ and J_max25_. Before flowering, the ability of N_area_, Chl_area_, and Car_area_ to explain the temporal variation of V_pmax25_ was limited, with all coefficients of determination (R^2^) below 30% (R^2^ = 0.05, 0.11, and 0.21, respectively), and none reached statistical significance (**Figures 4a, d, g**). After flowering, the correlation between V_pmax25_ and Chl_area_ improved slightly (R^2^ = 0.45, p< 0.05), but remained moderately weak. The correlation between V_pmax25_ and Car_area_ (R^2^ = 0.24) showed no significant change compared to that before flowering. Furthermore, the correlation between V_pmax25_ and N_area_ also showed a slight increase but remained extremely low (R^2^ = 0.14).

Chl_area_ was able to capture 81% of temporal variations in V_cmax25_ before flowering (R^2^ = 0.81, p< 0.0001) (**Figure 4e**), followed by N_area_ (R^2^ = 0.78, p< 0.0001) (**Figure 4b**), and Car_area_ (R^2^ = 0.62, p< 0.001) (**Figure 4h**). For the post-flowering stage, the slopes of Chl_area_ Vs. V_cmax25_, N_area_ Vs. V_cmax25_ and Car_area_ Vs. V_cmax25_ all became larger than those of the pre-flowering stage. The correlation of Chl_area_ Vs. V_cmax25_ was also the strongest (R^2^ = 0.74, p< 0.0001) compared to those of N_area_ Vs. V_cmax25_ (R^2^ = 0.50, p< 0.01) and Car_area_ Vs. V_cmax25_ (R^2^ = 0.52, p< 0.001) for the post-flowering stage.

Similarly, the correlations of J_max25_ with leaf biochemical variables (i.e., N_area_, Chl_area_, and Car_area_) also differed pre- and post-flowering. Car_area_ was able to capture 82% of temporal variations in J_max25_ before flowering (R^2^ = 0.82, p< 0.0001) (**Figure 4i**), followed by Chl_area_ (R^2^ = 0.80, p< 0.0001) (**Figure 4f**) and N_area_ (R^2^ = 0.57, p< 0.01) (**Figure 4c**). Also, the slopes of Chl_area_ Vs. J_max25_, N_area_ Vs. J_max25_ and Car_area_ Vs. J_max25_ all became larger than those of the pre-flowering stage due to the different seasonal variations of photosynthetic with biochemical variables (**Figure 2**). The correlation of Chl_area_ Vs. J_max25_ was also the strongest (R^2^ = 0.57, p< 0.001) compared to those of N_area_ Vs. J_max25_ (R^2^ = 0.54, p< 0.001) and Car_area_ Vs. J_max25_ (R^2^ = 0.42, p< 0.01) for the post-flowering stage. In sum, leaf photosynthetic pigments are better proxies for leaf photosynthetic capacity than N_area_ in maize during the pre- and post-flowering stages.

### 3.4 The sensitivity of V_cmax25_ to predictive variables

**Figure 5:**
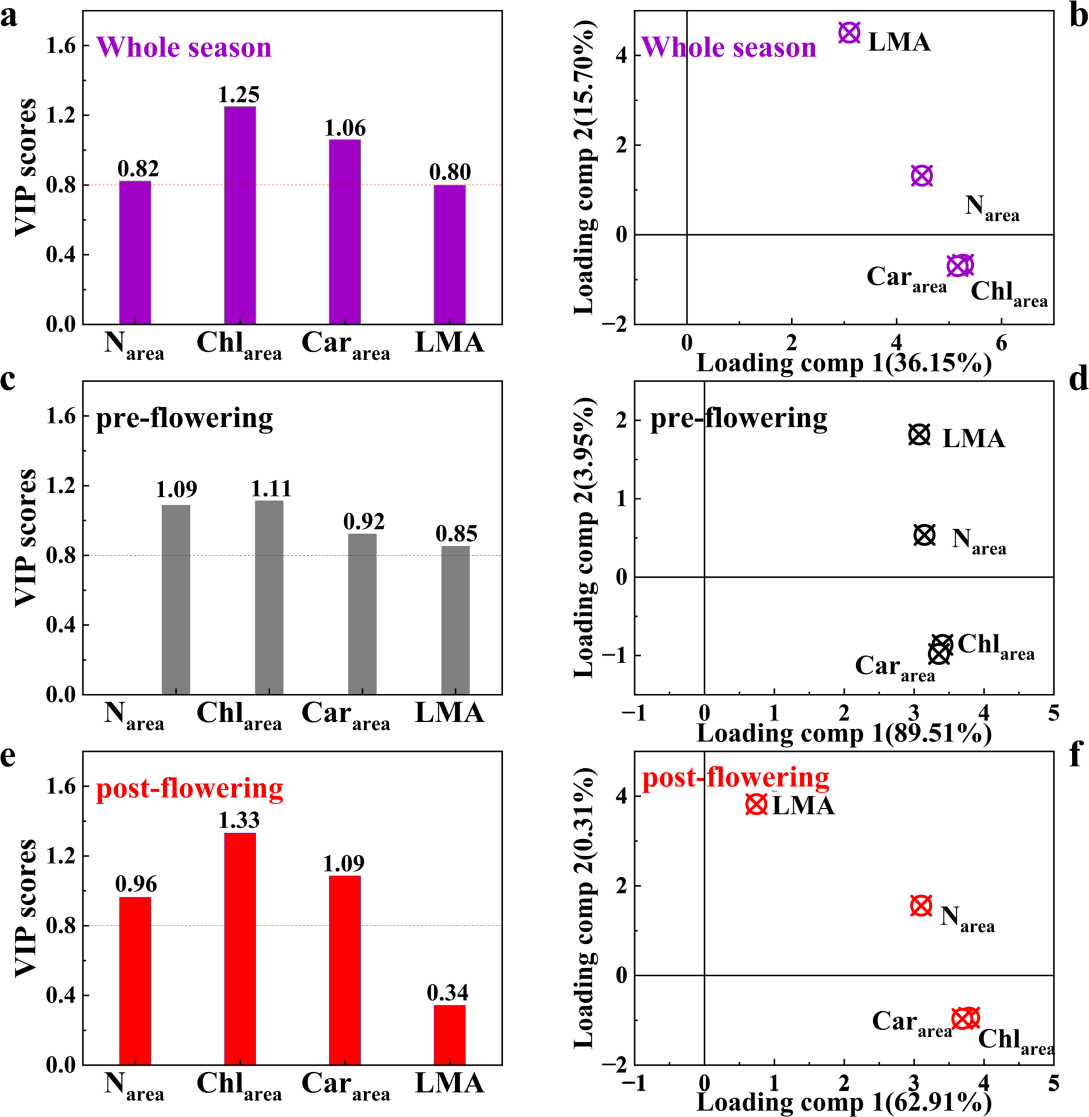
The VIP scores of leaf traits (N_area_, Chl_area_, Car_area_ and LMA) over V_cmax25_ for the whole season **(a)**, the pre-flowering stage **(c)** and the post-flowering stage **(e)**. The first and second loading components over V_cmax25_ for the whole season **(b)**, the pre-flowering stage **(d)** and the post-flowering stage **(f)**. The purple, grey and red colors represent the measurements at whole, pre- and post- flowering stages, respectively.

We computed the VIP scores and loadings to evaluate the relative contributions of four leaf traits (N_area_, Chl_area_, Car_area_, and LMA) for V_cmax25_ across different growth stages. In the VIP scores of the trait-based PLSR model, Chl_area_ was the primary contributor to V_cmax25_ prediction during the whole season, pre-flowering and post-flowering stages (VIP scores were 1.25, 1.11, and 1.33, respectively) (**Figures 5a, c, e**). The maize underwent vigorous vegetative growth before flowering, with leaf traits operating in a coordinated manner without significant functional divergence, resulting in minimal differences in VIP scores (VIP_Chl_ = 1.11, VIP_N_ = 1.09, VIP_Car_ = 0.92, VIP_LMA_ = 0.85) (**Figure 5c**). A greater divergence in the VIP scores of the leaf traits for V_cmax25_ was observed in the post-flowering stage compared with that in the pre-flowering stage. Moreover, the VIP score of LMA decreased significantly to 0.34 after flowering, which was markedly lower than those during the whole season (0.80) and the pre-flowering stage (0.85). This clear distinction is crucial for the precise identification of leaf traits governing V_cmax25_ across different growth stages.

Loading analysis revealed that the first loading component (LC1) accounted for 89.51%, 62.91%, and 36.15% of the V_cmax25_ variances during the pre-flowering, post-flowering, and whole season, respectively (**Figures 5d, f, b**). These results showed that the leaf traits exhibited a significantly stronger capacity to represent V_cmax25_ on the LC1 in the pre-and post-flowering stages compared with that of the whole season. With the second loading component (LC2) contributing significantly less to the variance in V_cmax25_ (pre-flowering: 3.95%; post-flowering: 0.31%; whole season: 15.70%) than LC1 in each respective stage, the LC1 effectively integrated the effects of the leaf traits on V_cmax25_ (**Figures 5b, d, f**). Additionally, Chl_area_ exhibited the highest positive loading on LC1 across different growth stages (**Figure 5**). The results consistently indicated that Chl_area_ is a more important driver for V_cmax25_ than other traits.

### 3.5 The predictive power of leaf trait combinations for V_cmax25_

**Figure 6:**
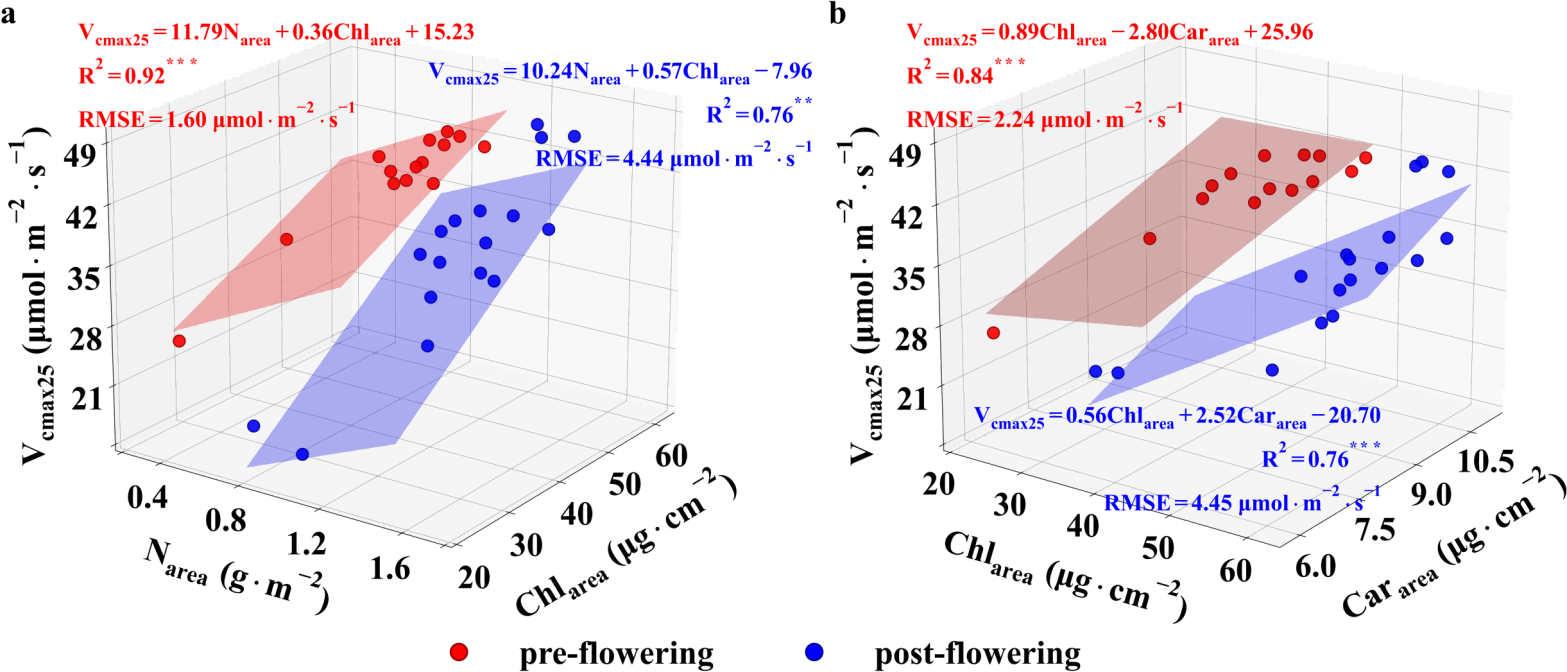
The relationship betweenV_cmax25_ and both N_area_ and Chl_area_ **(a)**, V_cmax25_ and both Chl_area_ and Car_area_ **(b)**. The red and blue points represent the measurements at pre- and post- flowering stages, respectively. *, ** and *** represent p< 0.01, p< 0.001, and p< 0.0001, respectively.

To improve the accuracy of estimating V_cmax25_, we analyzed the relationships between different combinations of leaf biochemical variables (i.e., F(N-Chl-Car), F(N-Chl), and F(Chl-Car)) and V_cmax25_ using multiple linear regression (**Figure 6**; **Table 2**). Before flowering, the combination based on leaf N_area_, Chl_area_, and Car_area_ consistently maintained the highest accuracy in estimating V_cmax25_ (R^2^ = 0.94, p< 0.0001, RMSE = 1.50 µmol·m^-2^·s^-1^), which was not significantly different from the combination based on leaf N_area_ and Chl_area_ (R^2^ = 0.92, p< 0.0001, RMSE = 1.60 µmol·m^-2^·s^-1^). However, F(Chl-Car) showed lower correlations with V_cmax25_ (R^2^ = 0.84, p< 0.0001, RMSE = 2.24 µmol·m^-2^·s^-1^) than F(N-Chl-Car) and F(N-Chl), respectively. After flowering, the coefficients of determination (R^2^) between all leaf trait combinations and V_cmax25_ showed a decrease but remained at relatively high levels (**Figure 6**; **Table 2**). In addition, the correlation strength between different leaf trait combinations and V_cmax25_ was consistently higher than that of individual leaf traits (Car_area_ and N_area_) during the pre- and post-flowering stages (**Figures 4**, **6**; **Table 2**). However, the relationship between leaf Chl_area_ and V_cmax25_ was slightly weaker than that of different leaf trait combinations. Thus, based on the empirical relationships shown in **Table 2**, the best equations could be used to estimate V_cmax25_ during the pre- and post-flowering (Equations 4, 5):

**Table 2:** Correlations of V_cmax25_ with N_area_, Chl_area_, and Car_area_.

F (N-Chl-Car)						
Growth Stage	R^2^	RMSE	a	b	c	d
Pre-flowering Post-flowering	0.94^***^ 0.78^***^	1.50 4.44	11.17 9.24	0.60 0.47	-2.131 2.26	22.96 -21.41

The regression modes follows the form V_cmax25_ = a×N_area_+b×Chl_area_+c×Car_area_+d, where a, b, and c represent the regression coefficients for N_area_, Chl_area_, and Car_area_, respectively, and d is the intercept term. RMSE (µmol m^-2^·s^-1^) is the root mean square error of estimated Vcmax25 against observations, *** indicates a significance level of p < 0.0001.

pre-flowering:

(4) $$V_{c\max25}\;=\;11.17N_{area}+0.60Chl_{area}-2.131Car_{area}+22.96$$

post-flowering:

(5) $$V_{c\max25}\;=\;9.24N_{area}+0.47Chl_{area}+2.26Car_{area}-23.41$$

## 4 Discussion

### 4.1 Impacts of seasonal variation in leaf nitrogen content

Leaf nitrogen plays a crucial role in the synthesis of photosynthetic proteins, and it is a primary regulatory factor in plant photosynthesis and growth (Khamis et al., 1990; Mu et al., 2016; Xu et al., 2012). This is because approximately 37% of leaf nitrogen is allocated to soluble protein, of which about 23% is Rubisco, about 9% is PEPc, about 6% is PPDK (Makino et al., 2003; Ghannoum et al., 2005; Mu et al., 2016). Therefore, a positive relationship has been observed between photosynthetic capacity and leaf nitrogen (Makino et al., 2003; Tazoe et al., 2006). Many TBMs constrain photosynthetic capacity by utilizing the relationship between leaf nitrogen content and V_cmax_ (Dietze, 2014). However, this relationship is regulated by environmental conditions (i.e., light regime, CO_2_ concentration, temperature, and leaf ontogeny). Therefore, the relationship between leaf nitrogen content and V_cmax25_ is not always stable. Similar to Miner and Bauerle (2019), our research also found weaker correlations between V_cmax25_ and N_area_ (R = 0.43) (**Figure 3**). This weaker correlation largely stemmed from the divergent seasonal variations of both variables (**Figure 2**).

The divergent seasonal variations of leaf N and V_cmax25_ was found in the middle of the growing season (**Figure 2**). The different trends may be attributed to nitrogen remobilization. During the vegetative growth stage, nitrogen is primarily allocated to the leaves and stems to support photosynthesis and promote crop growth (Ta and Weiland, 1992). During the reproductive growth stage, nitrogen remobilization was initiated to supply nutrients for the development of reproductive organs, as root activity and nutrient uptake generally decrease (Niu et al., 2010; Peng et al., 2010; Ciampitti et al., 2013; Gregersen et al., 2013). Nitrogen remobilization mainly occurs from the vegetative organs to the ear (Chen et al., 2015, 2016). Leaves and stems contributed 45% of total nitrogen remobilized into the ear, and 10% was contributed by the roots (Ta and Weiland, 1992). The primary nitrogen component remobilization from leaves is photosynthetic nitrogen, especially Rubisco, PPDK, and PEPc. This remobilization of nitrogen invariably results in a decreased photosynthetic rate. The nitrogen allocation patterns lead to a weaker relationship between leaf nitrogen and V_cmax25_ after flowering. Notably, the measured leaf nitrogen includes both photosynthetic and non-photosynthetic nitrogen, and its allocation may vary under different conditions (Mu et al., 2016; Dong et al., 2024; Liu et al., 2025). Additionally, the accurate estimation of leaf nitrogen from remote sensing data has faced persistent challenges due to spectral interference and scaling issues (Knyazikhin et al., 2013). Therefore, leaf nitrogen is not an ideal proxy for V_cmax25_ in large spatial scales.

### 4.2 Physiological basis for leaf chlorophyll content as a proxy for photosynthetic capacity

In this study, we found that Chl_area_ is a more accurate proxy for V_cmax25_ than N_area_ (**Figures 4**, **5**). The physiological basis of using Chl_area_ to estimate V_cmax25_ lies on the hypothesis of the coordination among different photosynthetic components. Chlorophyll is one part of component of light-harvesting proteins, its primary role is the capture and conversion of light energy to drive the electron transport (from Photosystem II to Photosystem I) reactions in photosynthesis (Mu and Chen, 2021; Nelson and Yocum, 2006). This transport completes the conversion of light energy to chemical energy, producing energy (ATP) and reducing power (NADPH) for carbon fixation reactions (Allen, 2002; Niinemets and Keenan, 2014). In carbon fixation stage, C4 crops initially fix CO_2_ in mesophyll cells by PEPc into C4 acids, which are then decarboxylated in the bundle sheath cells to supply CO_2_ for Rubisco. Compared to the energy requirements of C3 plants, the CO_2_ fixation in C4 plants needs additional ATP to maintain the CO_2_ concentration mechanism (Ogawa et al., 2023; Munekage and Taniguchi, 2016). Therefore, the CO_2_ fixation process in C4 plants has a higher energy demand. The photosynthesis begins with light energy, which is captured by chlorophyll molecules to drive the synthesis of NADPH and ATP to store chemical energy. There is a close relationship between the chlorophyll content and photosynthesis (Takashima et al., 2004; Tazoe et al., 2006). Meanwhile, chlorophyll and photosynthetic enzymes (particularly Rubisco) are regulated by leaf nitrogen. Plants optimize the allocation of leaf nitrogen between chlorophyll and Rubisco to maximize photosynthetic rates under given resource availabilities (Hikosaka and Hirose, 1997; Prentice et al., 2014). In contrast to the complex effects of leaf nitrogen allocation on the relationship between leaf nitrogen content and Rubisco during the growing season, the correlation between leaf chlorophyll and Rubisco content was robust overtime. Lu et al. (2022) has showed that strong linear relationship between Rubisco and chlorophyll relationships for C4 plants. In addition, the estimation of leaf chlorophyll from both canopy- and leaf-level reflectance spectra is more feasible (Croft et al., 2013; Qian and Liu, 2020; Xu et al., 2019), which is important for mapping V_cmax25_ across large spatial scales.

### 4.3 The importance of differentiating pre- and post-flowering stages for estimating leaf photosynthetic capacity from Chl_area_ in maize

Although we found that Chl is an effective proxy for V_cmax25_, the predictive power of leaf Chl_area_ varied across growth stage. This study revealed that the relationship between Chl_area_ and V_cmax25_ in maize leaves varies significantly with growth stages (**Figure 4**). The growth cycle of maize is typically divided into two distinct stages: vegetative growth stage (pre-flowering) and reproductive growth stage (post-flowering). The pre-flowering stage is a vegetative growth stage in which plants prioritize the construction of photosynthetic systems. Leaf chloroplasts serve as photosynthetic reaction centers, which are responsible for light harvesting and electron transport, the critical processes for generating ATP and NADPH to support vigorous vegetative growth (Allen, 2002). The amount of Rubisco in C3 plants accounts for 20-30% of leaf nitrogen content (Evans and Seemann, 1989; Makino, 2003), it is estimated to be only 5-9% in C4 plants (Schmitt and Edwards, 1981; Sage et al., 1987; Makino et al., 2003). This indicates that C4 plants can save substantial nitrogen. The nitrogen resources saved due to their lower Rubisco content are not fully offset by the nitrogen demand of C4 cycle enzymes and allow a greater nitrogen investment in the thylakoid components (i.e., thylakoid-membrane proteins associated with light harvesting, electron transport, and photophosphorylation) (Makino et al., 2003). The coordinated regulation of nitrogen allocation between Rubisco and thylakoid components aligns the chlorophyll-driven light energy supply with V_cmax25_. Thus, a strong correlation was observed between leaf Chl_area_ and V_cmax25_ before flowering, followed by that between leaf N_area_ and V_cmax25_. Notably, Chl_area_ and Car_area_ were more closely related to J_max25_ than to V_cmax25_ before flowering (**Figure 4**). Because J_max25_ was related to the capacity of the light-harvesting apparatus, which was determined by Chl_area_ and Car_area_ (Collatz et al., 1991; Sellers et al., 1992).

After flowering, the growth stage of maize shifts from vegetative to reproductive growth. Chl_area_ is still maintained at a relatively high level to support grain filling. In addition, the antioxidant function of carotenoids is enhanced to delay leaf senescence (Young, 1991), which may consequently cause chlorophyll to decline slowly. However, 60% to 85% of the nitrogen of vegetative organs was remobilized to the grain during the post-flowering stage. This nitrogen remobilization promotes the degradation of nitrogen components (i.e., PEPc, PPDK, Rubisco, thylakoid N), which are closely related to photosynthesis. This resulted in inconsistencies in the seasonal variation trends between V_cmax25_ and Chl_area_, and it also accounted for the decline in the correlation coefficient between Chl_area_ and V_cmax25_ during the post-flowering stage. Therefore, the relationship between V_camx25_ and Chl_area_ in maize is dependent on the growth stage. Distinguishing growth-stage dependent changes of leaf photosynthetic pigments (i.e. Chl_area_) are of great significance for the more accurate estimation of leaf photosynthetic capacity in maize.

## 5 Conclusions

In this study, we measured the seasonal variations in leaf photosynthetic capacity (V_pmax25_, V_cmax25_, and J_max25_), N_area_, and leaf photosynthetic pigments (i.e., Chl_area_ and Car_area_) in maize during the growing season of 2021. The major conclusions could be drawn as follows:

1. During the pre-flowering stage, the seasonality of leaf photosynthetic pigments was consistent with that of V_cmax25_ and J_max25_, but differed from V_pmax25_. Leaf photosynthetic pigments declined later than V_pmax25_, V_cmax25_, and J_max25_ during the post-flowering stage.
2. The relationships between leaf photosynthetic capacity and leaf biochemical traits exhibited significant growth-stage dependency, with leaf photosynthetic pigments (i.e. Chl_area_) showing the strongest correlation before and after flowering.
3. Leaf chlorophyll content serves as a reliable proxy for leaf photosynthetic capacity, particularly V_cmax25_.

These findings highlight that integrating leaf chlorophyll content with growth stage information can improve the accuracy of estimating photosynthetic capacity in maize, which has important implications for terrestrial biosphere models (TBMs) by enhancing the representation of photosynthetic capacity, thereby improving the simulation of carbon assimilation and crop productivity in farmland ecosystems. However, this study was limited to a single cultivar under specific environmental conditions. Future research should focus on validating these relationships across diverse maize genotypes and environmental conditions to refine model parameterization and predictive capabilities further.

## Data Availability

The original contributions presented in the study are included in the article/**Supplementary Material**. Further inquiries can be directed to the corresponding author.

## Supplementary material

Supplementary Figure 1 Modelled rate of CO_2_ assimilation as a function of intercellular CO_2_ concentration (C_i_) for the C4 photosynthetic pathway. V_pmax_ is the maximum PEP carboxylation rate, V_cmax_ is the maximum Rubisco carboxylation rate, J_max_ is the maximum electron transport rate, K_p_ is the Michaelis-Menten constant for CO_2_, V_pr_ is the PEP regeneration rate, and Tleaf is the leaf temperature. **(a–k)** respectively represent the measured data on the 190th, 203rd,206th, 214th,221st, 228th, 235th, 242nd, 252nd, 260th, and 267th days of 2021 at Yucheng Station.
