# Supplementary material for: Growth-stage dependent changes of leaf chlorophyll content as a proxy for photosynthetic capacity in maize
Source: Front Plant Sci. 2026 Mar 19;17:1758994. doi: 10.3389/fpls.2026.1758994 (PMC13044070; doi:10.3389/fpls.2026.1758994)
Supplement: Supplementary file 1 [file Table1.docx]

Supplementary Material

1. The equations of the C4 photosynthesis model

The leaf photosynthesis rate (A) of C4 species can be described as the minimum of two potential rates (Von Caemmerer, 2021) as:

| A = min (A_c_, A_j_) | (1-1) |
| --- | --- |

where A_c_ is the enzyme limited rate of CO_2_ assimilation and A_j_ is the electron transport limited rate of CO_2_ assimilation.

**A_c_ can be expressed as:**

| A_c_ = min {(V_cmax_ - R_d_), (V_p_ – R_m_ + g_bs_C_m_)} | (1-2) |
| --- | --- |

where V_cmax_ is the maximum Rubisco carboxylation rate. R_d_ is the rate of mitochondrial respiration not associated with photorespiration. R_m_ is the mitochondrial respiration occurring in the mesophyll. Usually, the mesophyll CO_2_ concentration (C_m_) was set equal to the intercellular CO_2_ concentration (C_i_) and mesophyll resistance to CO_2_ diffusion was ignored. g_bs_ is the conductance to CO_2_ leakage and is determined by the properties of the bundle sheath cell wall. V_p_ can be expressed as:

| $\text{V}_{\text{p}}\text{ }\text{=}\text{ }\text{min}\text{ }\text{(}\frac{\text{C}_{\text{m}}\text{V}_{\text{pmax}}}{\text{C}_{\text{m}}\text{+}\text{K}_{\text{p}}}\text{, }\text{V}_{\text{pr}}\text{)}$ | (1-3) |
| --- | --- |

V_pmax_ is the maximum PEP carboxylation rate, and K_p_ is the Michaelis-Menten constant for CO_2_. V_pr_ is the rate of PEP regeneration, which is mainly controlled by the PPDK enzyme.

**A_j_ can be expressed as:**

| A_j_ = min {($\frac{\text{z}}{\text{2}}\text{xJ}\text{-}\text{R}_{\text{m}}\text{+}\text{g}_{\text{bs}}\text{C}_{\text{m}}$), ($\frac{\text{z}}{\text{3}}\left[ \text{1-x} \right]\text{J-}\text{R}_{\text{d}}$)} | (1-4) |
| --- | --- |

where x equals 0.4, and z relates linear electron flow J to the rate of ATP production. J is the potential whole-chain linear electron transport, and it can be expressed:

| $\text{J}\text{ }\text{= }\frac{\left( \text{Q}_{\text{p2}}\text{+}\text{J}_{\text{max}} \right)\text{-}\sqrt{{\text{(}\text{Q}_{\text{p2}}\text{+}\text{J}_{\text{max}}\text{)}}^{\text{2}}\text{-4}\text{Q}_{\text{p2}}\text{J}_{\text{max}}}}{\text{2ɵ}}$ | (1-5) |
| --- | --- |

Q_p2_ is the photosynthetically useful light absorbed by PSII. J_max_ is the maximum electron transport, and ɵ is an empirical curvature factor.

2. The simulation equations for Acp, Acr, and Aj based on estimated V_pmax_, V_cmax_, and J_max_

**Acp (photosynthetic rate limited by PEP carboxylase) can be expressed:**

| $\text{Acp}\text{ }\text{= min}\text{ }\text{(}\frac{\text{C}_{\text{i}}\text{V}_{\text{pmax}}}{\text{C}_{\text{i}}\text{+}\text{K}_{\text{p}}} \text{-}{\text{ }\text{R}}_{\text{m}}\text{ }\text{+}\text{ }\text{g}_{\text{bs}}\text{C}_{\text{i}}\text{, }\text{V}_{\text{pr}} \text{-}\text{ }\text{R}_{\text{m}}\text{ }\text{+}\text{ }\text{g}_{\text{bs}}\text{C}_{\text{i}}\text{)}$ | (2-1) |
| --- | --- |

where C_i_ is the intercellular CO_2_ concentration, V_pmax_ is the maximum PEP carboxylation rate, K_p_ is the Michaelis-Menten constant for CO_2_, and V_pr_ is the rate of PEP regeneration. R_m_ is the mitochondrial respiration occurring in the mesophyll, with a value of 0.5R_d_. g_bs_ is the conductance to CO_2_ leakage, with a value of 0.003.

**Acr (photosynthetic rate limited by Rubisco) can be expressed：**

| $\text{Acr}\text{ }\text{= }\text{V}_{\text{cmax}}\text{-}\text{R}_{\text{d}}$ | (2-2) |
| --- | --- |

where V_cmax_ is the maximum Rubisco carboxylation rate. R_d_ is the rate of mitochondrial respiration not associated with photorespiration, with a value of 1.0 µmol·m^-2^·s^-1^.

**Aj (photosynthetic rate limited by electron transport) can be expressed：**

| $\text{Aj = min (}\frac{\text{z}}{\text{2}}\text{x}\frac{\left( \text{Q}_{\text{p2}}\text{+}\text{J}_{\text{max}} \right)\text{-}\sqrt{{\text{(}\text{Q}_{\text{p2}}\text{+}\text{J}_{\text{max}}\text{)}}^{\text{2}}\text{-4}\text{Q}_{\text{p2}}\text{J}_{\text{max}}}}{\text{2ɵ}}\text{-}\text{R}_{\text{m}}\text{+}\text{g}_{\text{bs}}\text{C}_{\text{i}}\text{, }\frac{\text{z}}{\text{3}}\left( \text{1-x} \right)\frac{\left( \text{Q}_{\text{p2}}\text{+}\text{J}_{\text{max}} \right)\text{-}\sqrt{{\text{(}\text{Q}_{\text{p2}}\text{+}\text{J}_{\text{max}}\text{)}}^{\text{2}}\text{-4}\text{Q}_{\text{p2}}\text{J}_{\text{max}}}}{\text{2ɵ}}\text{-}\text{R}_{\text{d}}\text{)}$ | (2-3) |
| --- | --- |

where x equals 0.4; z relates linear electron flow J to the rate of ATP production, with a value of 1.25. ɵ is an empirical curvature factor, with a value of 0.7. Q_p2_ is the photosynthetically useful light absorbed by PSII, and is determined by PPDF. J_max_ is the maximum electron transport.

**Note:** the parameters V_pmax_, V_cmax_, J_max_, V_pr_, and K_p_ are estimated by the “minpack.lm” and “Metrics” packages (<https://zhujiedong.quarto.pub/photosynthesis-school-2023/#/c4-%E6%A4%8D%E7%89%A9%E6%89%8B%E5%86%99%E4%BB%A3%E7%A0%81%E5%8F%8A%E4%BD%9C%E5%9B%BE>).
