# Supplementary figures and images for: Growth-stage dependent changes of leaf chlorophyll content as a proxy for photosynthetic capacity in maize
Source: Front Plant Sci. 2026 Mar 19;17:1758994. doi: 10.3389/fpls.2026.1758994 (PMC13044070; doi:10.3389/fpls.2026.1758994)

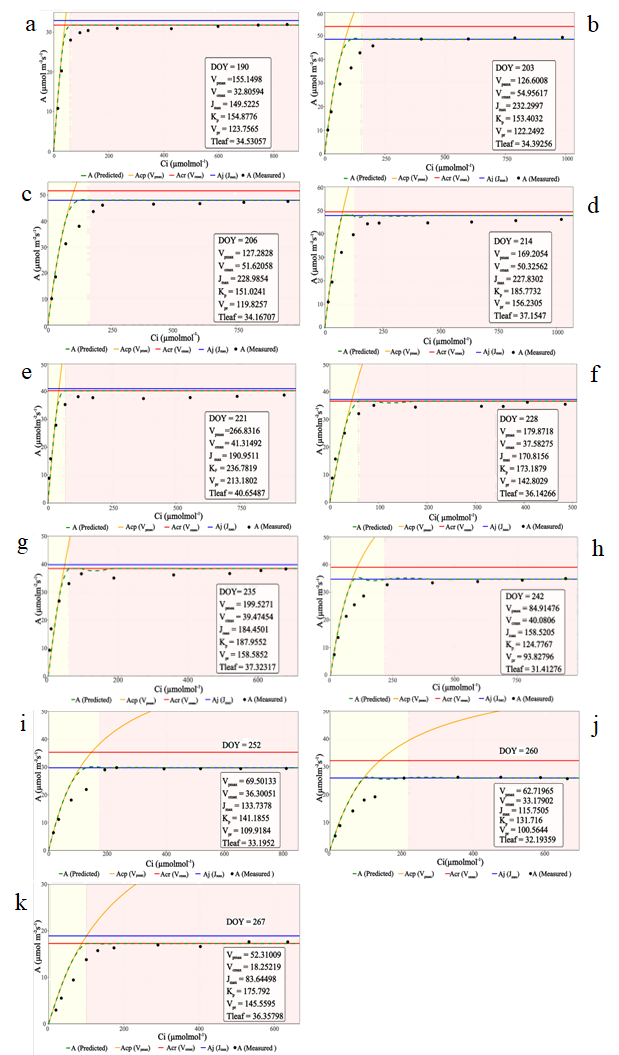

Supplement: Supplementary Figure 1 — Modelled rate of CO2 assimilation as a function of intercellular CO2 concentration (Ci) for the C4 photosynthetic pathway. Vpmax is the maximum PEP carboxylation rate, Vcmax is the maximum Rubisco carboxylation rate, Jmax is the maximum electron transport rate, Kp is the Michaelis-Menten constant for CO2, Vpr is the PEP regeneration rate, and Tleaf is the leaf temperature. (a–k) respectively represent the measured data on the 190th, 203rd,206th, 214th,221st, 228th, 235th, 242nd, 252nd, 260th, and 267th days of 2021 at Yucheng Station. [file Image1.png]
